# Characterization of carbapenem-resistant Acinetobacter baumannii strains isolated from hospitalized patients in Palestine

**DOI:** 10.1186/2047-2994-4-S1-P136

**Published:** 2015-06-16

**Authors:** R Handal, L Qunibi, I Sahouri, M Juhari, H Marzouqa, M Hindiyeh

**Affiliations:** 1 Laboratory, Caritas Baby Hospital, Bethlehem, Palestinian Territory, Occupied; 2 Laboratory, Alia Hospital, Hebron, Palestinian Territory, Occupied; 3 Laboratory, Hussein Hospital, Bethlehem, Palestinian Territory, Occupied; 4 Laboratory, Rafidia Hospital, Nablus Palestinian Territory, Occupied; 5 Medicine, Caritas Baby Hospital, Bethlehem, Palestinian Territory, Occupied

## Introduction

The increase in the incidence of hospital acquired infections due to *A. baumannii* (MDR-AB) mandates characterizing the strains circulating in Palestinian hospitals.

## Objectives

Determine the antibiogram of the MDR-AB. Identify the genes responsible for the carbapenem and the aminoglycosides resistance. Identify the presence of the two virulence genes OmpA and epsA. Determine the strains types of the MDR-AB isolated from Palestinian hospitals.

## Methods

72 single patients MDR-AB collected from all over Palestine, except Gaza, were included in the study. The CLSI guidelines were followed to determine the antibiogram of the isolates. The presence of the carbapenem resistance genes *bla*_OXA-58,_*bla*_OXA-23_, *bla*_OXA-24_, *bla*_KPC_, *bla*_NDM_ and the aminoglycoside resistance genes *aphA*6 and *aphA*1 were determined by PCR. Moreover, the two *A. baumannii* virulence genes OmpA and epsA, were evaluated by PCR. Finally MLST was performed on 13 isolates to determine the Strain Type (ST) of the isolates.

## Results

All the isolates were resistant to all the β–lactam antibiotics including the carbapenems. Of the 72 isolates, 77.9% positive for *bla*_OXA-23,_ 14.7% positive for *bla*_OXA-24_, 4.4% positive for *bla*_OXA-58_. In addition, 5.88% and 0% were positive for *bla*_NDM_ and *bla*_KPC,_ respectively. Moreover, of the 72 isolates none were positive for *aphA*6 gene while 92% were positive to the *aphA*1 gene. The only two antibiotics that showed a non-resistant profile were colistin sulfate (78%) and tigecycline (95%). 98.5% of the isolates possessed the OmpA biofilm producing virulence gene. Finally, MLST of 13 isolates revealed that more than one strain of *A. baumannii* is circulating in the Palestinian hospitals, 7 isolates ST 208 (53.8%), 2 isolates ST 218 (15.4%), 1 isolates ST231 (7.7%), 1 isolates ST348 (7.7%) and 2 isolates new ST (15.4%).

## Conclusion

The detection of these extremely drug resistant pathogens in Palestine was a strong reminder of the importance of mandating that the infection control programs in all the hospitals must be active in order to reduce the spread of these deadly pathogens.

## Disclosure of interest

None declared.

